# Type 2 Diabetes Mellitus Provokes Rat Immune Cells Recruitment into the Pulmonary Niche by Up-regulation of Endothelial Adhesion Molecules

**DOI:** 10.34172/apb.2022.019

**Published:** 2020-10-19

**Authors:** Eghbal Zarafshan, Reza Rahbarghazi, Jafar Rezaie, Mohammad Reza Aslani, Shirin Saberianpour, Mahdi Ahmadi, Rana Keyhanmanesh

**Affiliations:** ^1^Department of Physiology, Faculty of Medicine, Tabriz University of Medical Sciences, Tabriz, Iran.; ^2^Stem Cell Research Center, Tabriz University of Medical Sciences, Tabriz, Iran.; ^3^Department of Applied Cell Sciences, Faculty of Advanced Medical Sciences, Tabriz University of Medical Sciences, Tabriz, Iran.; ^4^Solid Tumor Research Center, Research Institute for Cellular and Molecular Medicine, Urmia University of Medical Sciences, Urmia, Iran.; ^5^Ardabil Imam Khomeini Educational and Clinical Hospital, Ardabil University of Medical Sciences, Ardabil, Iran.; ^6^Vascular and Endovascular Surgery Research Center, Mashhad University of Medical Sciences, Mashhad, Iran.; ^7^Drug Applied Research Center, Tabriz University of Medical Sciences, Tabriz, Iran.

**Keywords:** Type 2 Diabetes Mellitus, Lungs, Vascular Cell Adhesion Molecules, Inflammation, Nitrosative Stress

## Abstract

***Purpose:*** Diabetes mellitus, especially type 2, is conceived as a devastating chronic metabolic disease globally. Due to the existence of an extensive vascular network in the pulmonary tissue, it is suggested that lungs are sensitive to the diabetic condition like other tissues. This study was designed to address the possible effect of type 2 diabetes mellitus on the promotion of pathological changes via vascular injury.

***Methods:*** Sixteen male Wistar rats were randomly allocated to the two of control and T2D groups. To induce type 2 diabetes (T2D), rats were received high-fat and a single dose of streptozotocin (STZ). On week 12, rats were euthanized and lungs samples were taken. Using hematoxylin and eosin (H&E) staining, the pathological changes were monitored. The expression of intercellular adhesion molecule (ICAM-1) and vascular cell adhesion molecule 1 (VCAM-1), and interleukin 10 (IL-10) was monitored using real-time PCR assay. The level of tumor necrosis factor-α (TNF-α) was detected using ELISA assay. Nitrosative stress was monitored using the Griess assay.

***Results:*** Pathological examination in bronchoalveolar discharge revealed the existence of mild to moderate interstitial bronchopneumonia and increased neutrophilic leukocytosis compared to the control. Enhanced ICAM-1 and VCAM-1 expression and suppression of IL-10 was found using real-time PCR analysis (*P* < 0.05). The levels of TNF-α and NO were increased with diabetic changes compared to the control rats (*P* < 0.05).

***Conclusion:*** T2D could promote pulmonary tissue injury via the production of TNF-α and up-regulation of vascular ICAM-1 and VCAM-1. The inflammatory status and vascular ICAM-1 and VCAM-1 increase immune cell recruitment into the pulmonary niche.

## Introduction

Diabetes mellitus, especially type 2, is conceived as a metabolic disorder that is prevalent in adults.^1^ It has been estimated that there are more than 500 million prevalent cases of type 2 diabetes (T2D) in 2018 with a heavy socio-economical burden.^2^ Prolonged hyperglycemic fluctuations in T2D provide profound detrimental outcomes in multiple organs.^3^ In support of previous data, high diabetic subjects mortality, reaching 80%, correlated with macro and microvascular complications.^4^ Considering the existence of a dense vascular network and high collagen and elastin fibers content inside pulmonary parenchyma, these features make the lungs vulnerable to pro-inflammatory conditions during chronic hyperglycemia.^3^ Of note, most efforts have been focused to assess the detrimental effects of T2D on the central nervous system, retina vessels, and hepatic, testicular tissue and cardiovascular system as well.^4-7^ Data from clinical observations and examinations revealed the reduction in functional vital capacity of T2D lungs coincided with abnormal pulmonary histopathology.^8-10^ Along with these changes, the number of apoptotic endothelial cells (ECs) increased by the activation of Caspase-related signaling cascades.^11^ In a recent cross-sectional study, it was shown that the increase of acute-phase proteins and pro-inflammatory mediators such as tumor necrosis factor-α (TNF-α) and C-reactive protein (CRP) in non-controlled T2D candidates led to lowerforced vital capacity **(**FVC) and forced expiratory volume in one second (FEV1) values.^12^ Similar to studies targeting diabetes in human medicine, it was elucidated the promotion of profound long-term diabetic effects contributed to the induction of progressive inflammatory and fibrotic changes in the lung tissue of *streptozotocin* (*STZ)-*induced *diabetic rats.*^13,14^ Despite the existence of the huge amount of clinical data related to chronic hyperglycemia on pulmonary parenchyma, it seems that the putative effects of diabetic conditions have been neglected in the context of pulmonary ECs from both basic and clinical studies.^9,13^ Therefore, it is highly recommended to measure the extent of pulmonary ECs involvement during the chronic hyperglycemic condition. Calling attention, the majority of animal studies only explored the detrimental effects of diabetic condition induced STZ on pulmonary tissue which is not completely comparable to the T2D in *in vivo* condition.^14,15^ As expected, the direction and recruitment of immune cells toward the pulmonary niche are mediated by the close interaction of immune cells with ECs.^16,17^ To our knowledge, there are not enough data related to the dynamic of adhesion molecules mainly intercellular adhesion molecule-1 (ICAM-1) and vascular cell adhesion molecule 1 (VCAM-1) in T2D pulmonary ECs. In line with these comments, the current experiment was conducted to assess the underlying mechanisms that participated in the promotion of pulmonary inflammation via adhesion molecules in T2D rats induced by STZ and high-fat diet. The levels of ICAM-1, VCAM-1and pro-inflammatory cytokines will be measured in pulmonary tissue after the promotion of T2D. Data from this study could help us in the understanding of pulmonary EC-immune cell reciprocal interaction seen during the diabetic condition and develop novel therapies based on the modulation of adhesion molecules.

## Material and Methods

### Animal ethics

The animal experiments described in this study were performed under the recommendations in the Guide for the Care and Use of Laboratory Animals (NIH Publication No. 85- 23, revised 1996).

### Experimental groups

To perform the current experiment, sixteen male Wistar rats (initially weighing 190–210 g) were enrolled. All animals were kept in standard cages (4 rats per cage) for 10 days. Animals were maintained at 22 ± 2°C, with a constant humidity of 45–55%, on a 12-hour light-dark cycle (07.00 on/19.00 off) with free access to food and tap water, before the manipulation. Animals were randomly allocated into two experimental groups (each in 8 rats) as follows: Control rats received only regular chow and tap water (C group); Diabetic rats received high-fat diets (HFD; 48% carbohydrate, 22% fat and 20% protein) and a low dose of STZ (T2D group).

### Induction of T2D in rats

T2D was induced using the HFD feeding for consequent 4 weeks followed by a single intraperitoneal (i.p.) injection of a low dose of STZ (35 mg/kg; Sigma Aldrich, Germany). After 3 days of STZ injection, a blood sample of the tail vein was obtained and non-fasting plasma glucose was measured using a digital glucometer (Norditalia Elettro medica liSrL., Italy). Rats with the non-fasting plasma glucose ≥ 300 mg/dL were considered as diabetic status.^18,19^ After induction of T2D, rats were received HFD for the next 8 weeks. Thereafter, all animals were euthanized 8 weeks after diabetes confirmation (for molecular and histological analysis of lung tissues. Control rats were given regular food and water through the experiments.

### Glucose tolerance test (GTT)

Before sacrifice (12^th^ week), a glucose tolerance test was performed after oral glucose administration (1 g/kg). Before the GTT assay, animals had no access to food for 12 hours. Blood samples were obtained for glucose measurements from the tail vein before and again at time points 0, 30, 60, 90, and 120 minutes after glucose administration.^19^

### Bronchoalveolar lavage fluid (BALF) preparation

Immediately after pectoral incision, BALF was sampled by five consecutive 1 mL instillations of normal saline by a *catheter* connected to each trachea. Then; we diluted the same volume of BALF with Turk solution and total white blood cells were counted by using a *Neubauer slide. To calculate the content of neutrophils in BALF, samples were centrifuged at*2500 gfor 4°C* at*10 minutes, smeared on glass slides, and stained with Wright-Giemsa solution as previously described.^20^

### Nitric oxide measurement

Supernatants collected after BALF centrifugation was used for determining nitric oxide (NO) content in each group. For this propose, the content of NO was measured by using Griess reagent. This substrate converts unstable NO into a more stable nitrogen product namely nitrite. Further biochemical reaction contributes to the formation of nitrous acid in an acidic condition. The addition of sulfanilamide to this condition forms a diazonium salt and azo dye, respectively. The intensities of optical densities were measured at 540 nm by using a microplate reader (ELx808; BioTek; USA). NO levels were expressed as μmol/L after comparing them with the sodium nitrite standard curve.

### Measurement of TNF-α level in BALF by ELISA method

The TNF-α level was measured in BALF using rat ELISA kits (Glory science co. Ltd, USA) according to the manufacturer’s instructions.

### Real-time PCR analysis

The expression levels of *interleukin 10 (IL-10)*, ICAM-1, and VCAM-1 were determined by quantitative real-time PCR assay.^21^ Primers were designated by Gene-Runner software (Ver. 3.05) and outlined in Table 1. To run gene expression analysis, a part of left lung tissue from rats was immediately frozen in -196°C nitrogen solution, smashed roughly, and total RNA isolated using YTA total RNA extraction mini kit (YTA, Iran). The integrity of isolated RNAs was detected by a Nanodrop^®^ ND-1000 UV-VIS Spectrophotometer (Thermo Scientific, Wilmington DE 19810 USA) and Standard agarose gel electrophoresis (Supplementary file 1, Figure S1). RNAs were reverse-transcribed into cDNA by using the cDNA Synthesis Kit (YTA, Taiwan). Quantitative real-time PCR was done by using the SYBR Green Master Mix (YTA, Taiwan) and Rotor-Gene 6000 apparatus (Corbett Life Science, Australia). The expression of target genes was normalized to control housekeeping gene GAPDH and values were expressed as relative fold changes using the 2-^∆∆CT^ method.

**Table 1:** Primer list used for miRNAs

**Gene**	**Primer sequence (5'-3')**	
	**Forward**	**Reverse**
VCAM-1	GTG TGT GAA GGA GTG AAT CTG G	CCA ACA GCA GCA CAT GTC AGA A
ICAM-1	TGG AGG TGA CTG AGA AGT TGG	CAC AGT TAC TTG GTC CCC TTC
*IL-10*	TGAGAATAAAAGCAAGGCAGTGG	GTAGGCTTCTATGCAGTTGATGA
GAPDH	TTG CCA TCA ACG ACC CCT TCA	AGC ACC AGC ATC ACC CCA TTT

### Histological evaluation

To assess the pathological effect of T2D on the pulmonary niche, right lung lobes were sampled and fixed in 10% neutral buffered formalin (37%, Merck, Darmstadt; Germany). Thereafter, samples were embedded in paraffin blocks and cut into 4-µm thick sections. Slides were stained with hematoxylin and eosin (H&E) solution and visualized by light microscopy (Model: BX41; Olympus; Japan).^16^ The existence of tissue damages such as mild interstitial pneumonitis and bronchiolar epithelium degeneration was investigated by an expert pathologist.

### Data analysis

All quantitative results were analyzed using a student *t*test and presented as mean ± SEM. Statistical significance was set at *P* < 0.05.

## Results

### GTT** and** serum glucose concentrations confirmed diabetic changes

**Figure 1:**
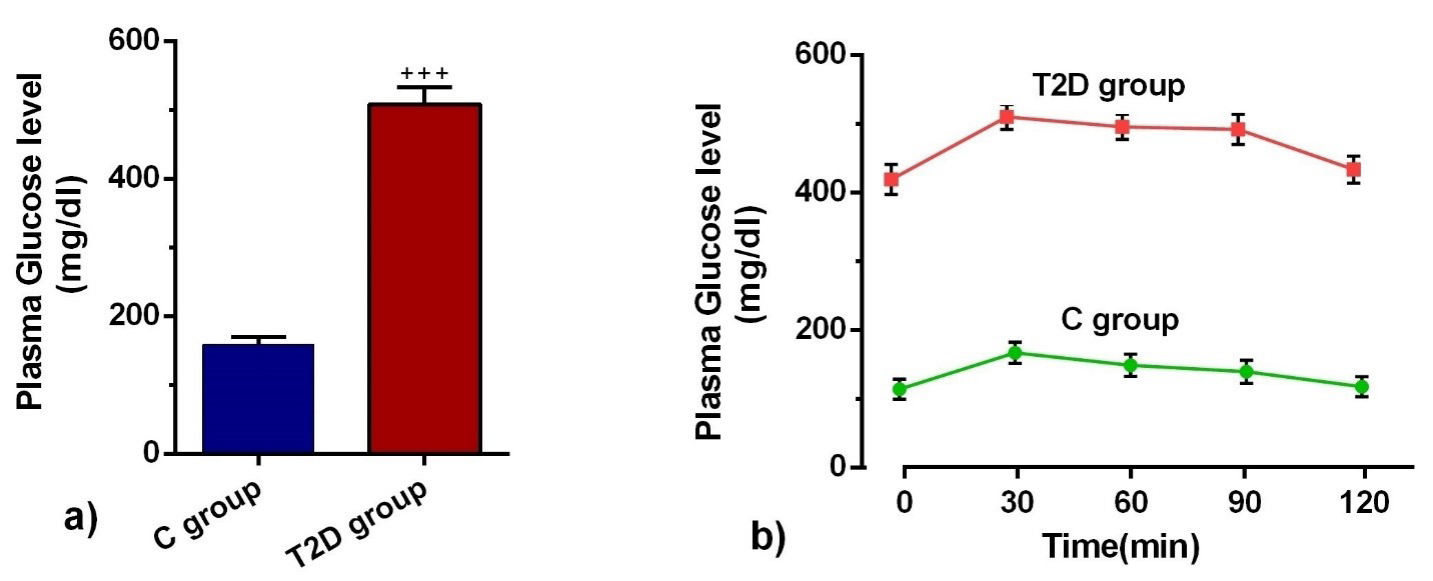
BS levels (a) and GTT assay (b) in control and diabetic groups (n = 8). Bars represent the mean ± SEM. Statistical differences between control and diabetic groups: and + + + *P* < 0.001.

We measured BS glucose levels and performed a GTT assay to confirm diabetic changes in STZ-administrated rats. Based on our data, BS levels were increased 3 days after STZ administration in T2D rats compared to the control group (Figure 1a). Also, GTT on weeks 12 showed impaired glucose tolerance activity in T2D rats compared to the healthy control counterpart, showing pancreatic tissue insufficiency (Figure 1b). The data confirmed that our protocol successfully induced T2D.

### Total leukocyte count and neutrophil percentage were increased in BALF of T2D group

**Figure 2:**
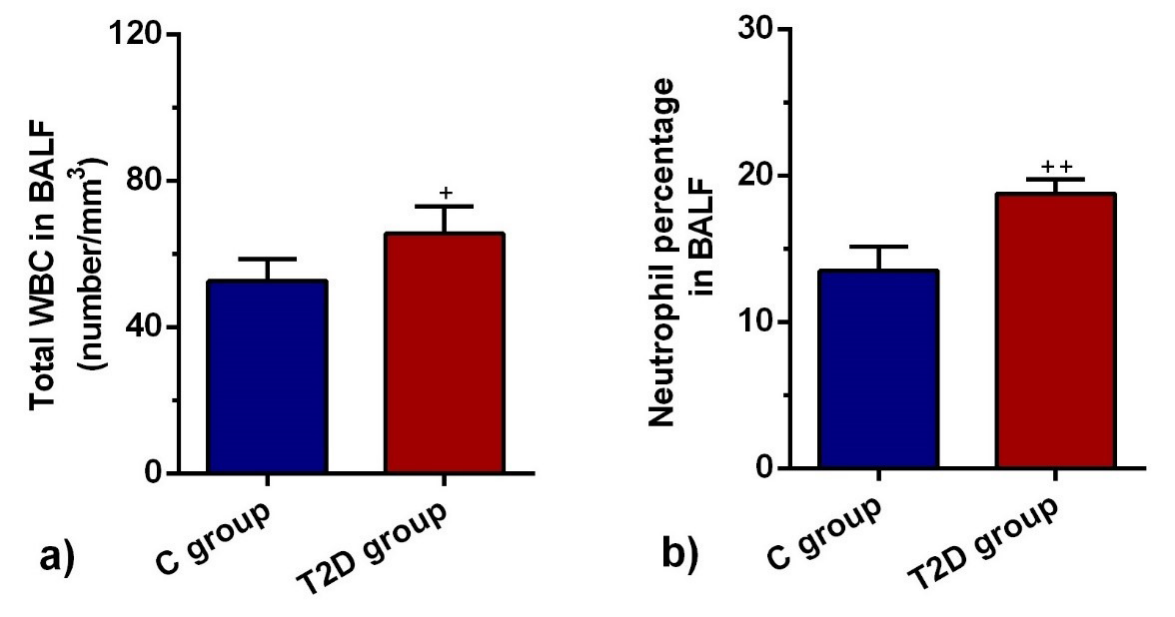
The mean value of total leukocyte (a) and neutrophil percentage (b) in BALF of control and diabetic rats (n = 8). Bars represent the mean ± SEM. Statistical differences between control and diabetic groups: + *P* < 0.05 and + + *P* < 0. 01.

The mean value of the total leukocyte population was significantly higher in BALF of the T2D group compared to the C group (*P* < 0.05; Figure 2a). There was a significant increase in the neutrophil percentage of the T2D group in comparison with the C group (*P* < 0.01; Figure 2b). The data showed that the promotion of T2D in rats induced inflammatory response and neutrophilic leukocytosis, showing acute pathological response in diabetic lungs.

### T2D contributed to the induction of ICAM-1 and VCAM-1 and reduction of IL-10 expression

**Figure 3:**
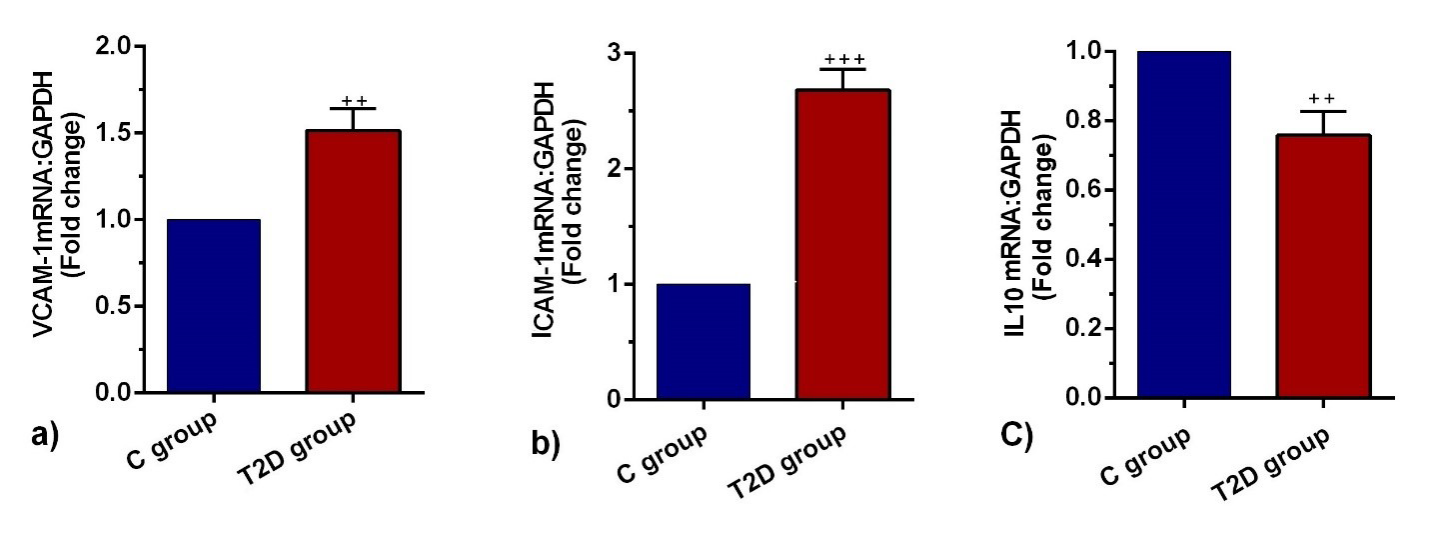
Measuring the expression of *ICAM-1*(a), *VCAM-1*(b), and IL-10 (c) mRNA in the lung tissues of control and diabetic rats (n = 8). Bars represent the mean ± SEM. Statistical differences between control and diabetic groups: + + + *P* < 0.001.

The promotion of endothelial surface adhesion molecules such as ICAM-1 and VCAM-1 promoted immune cell tethering and trans-endothelial migration to the inflammatory sites. Based on our analysis, VCAM-1 and ICAM-1 mRNAs were significantly increased in T2D pulmonary ECs compared to the control group (*P* < 0.01 and *P* < 0.001 respectively;Figure 3a, b). The expression levels of IL-10 in the T2D group were significantly lower than the C group (*P* < 0.01; Figure 3c). The up-regulation of VCAM-1 and ICAM-1 with simultaneous suppression of IL-10 showed active inflammation and vascular complication in diabetic niche.

### T2D condition initiated nitrosative stress in pulmonary tissue

**Figure 4:**
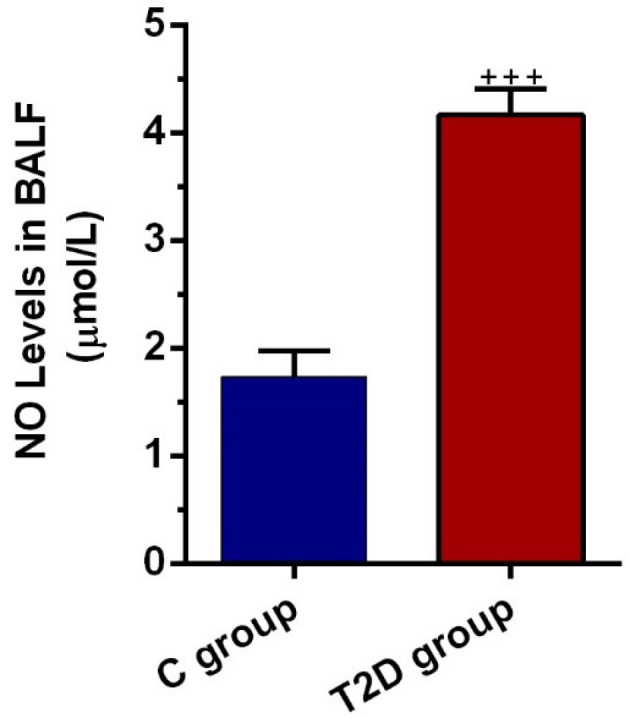
.NO levels in BALF of control and diabetic rats (n = 8). Bars represent the mean ± SEM. Statistical differences between control and diabetic groups: + + + *P* < 0.001.

NO levels in BALF were also determined to show the level of nitrosative stress in epithelial cells located at the pulmonary conduit. Data highlighted the increase of NO level in BALF from T2D rats compared to the control group (*P* < 0.001; Figure 4). We found an approximately 2.2-fold increase in BALF NO content in diabetic condition, showing nitrosative stress under the onset of chronic diabetic changes. Along with these data, the increase of NO in BALF could reflect the levels of bronchiolar epithelial cell injury in the rat model of T2D.

### T2D condition increased the BALF level of TNF-α

**Figure 5:**
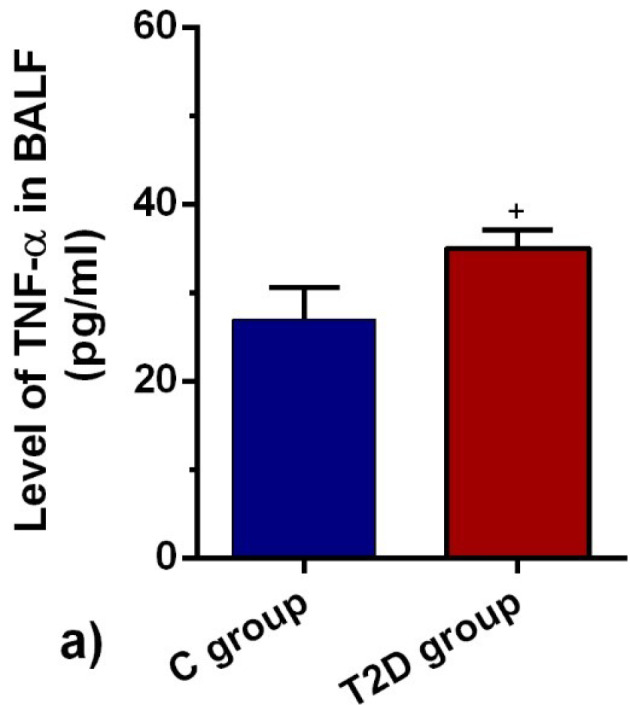
TNF-α in BALF of control and diabetic rats (n = 8). Bars represent the mean ± SEM. Statistical differences between control and diabetic groups: + *P* < 0.05.

The dynamic production of TNF-α highlighted the activation/inhibition of inflammatory cells in the target tissue. ELISA showed that the BALF level of TNF-α was drastically different in the diabetic group in comparison with the control rats (*P* < 0.05; Figure 5). Therefore, the reduction of IL-10 coincided with the increase of TNF-α exhibited active inflammatory status in the diabetic niche.

### Pulmonary pathological changes were achieved during the prolonged diabetic condition

**Figure 6:**
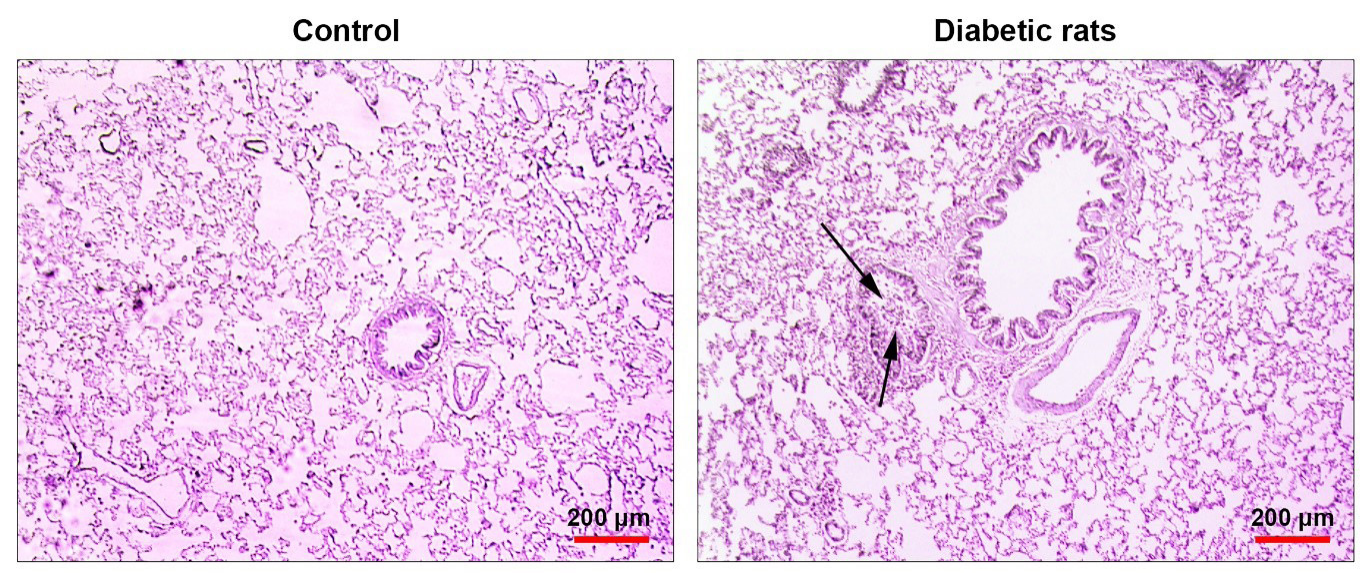
Bright-field images from lung tissue sections stained with H&E solution. Diabetic lungs are evident with mild interstitial pneumonitis and bronchiolar epithelium degeneration (black arrows). These data show chronic structural changes in Diabetic rats.

To confirm pathological changes in diabetic pulmonary tissue, we performed a histopathological examination on lung tissues of either control or diabetic rats. Bright-field microscopic imaging revealed a mild interstitial pneumonitis and bronchiolar epithelium degeneration in lung tissues of diabetic rats compared to the control rats. The pattern of chronic pathological changes in diabetic rats confirmed the pathological effect of T2D on a pulmonary niche (Figure 6).

## Discussion

Due to changes in lifestyle, reduced mobility, and eating habits, the emergence of distinct metabolic disorders mainly T2D has been increased.^22^ At molecular and cellular levels, T2D has the potential to promote various pathological changes contributing to tissue insufficiency and malfunction.^23^ In this regard, all tissues are sensitive, if not on the same levels, to glucose changes during long-term hyperglycemic conditions.^24^ It is logical to hypothesize that any disturbances in the function of blood vessels nourishing different tissues promote pathological changes.^25^ Regarding spirometric reports from the clinical setting, there is a close relationship between lung dysfunction and the occurrence of T1D and T2D.^8-10,12^ Up to the present time, the most of efforts have been focused on the discovery of T2D effects on cardiovascular tissues, brain, liver, and testes while pulmonary tissue, if not completely, undergo some pathological changes and adverse effects of T2D have not been fully addressed on this system.^3-7^

Of the most notably, pulmonary tissues encompass a large number of micro- and macro-vascular beds with a unique structure that actively participates in the gas interchange between pneumocytes and ECs.^3,21,25^ Along with these statements, the existence of large contents of collagen and elastin fibers predisposes pulmonary tissue to diabetic changes.^3^ Previous analysis of the histological slides revealed an increase in the extracellular matrix, as evidenced by the presence of fibrosis, increase thickness of the alveolar-capillary membrane, hyperplasia of the capillary endothelium in the lung tissue of STZ-induced diabetic animal models.^15^ Recent data pointed out that the combination of high-fat diets and STZ in rats could induce chronic diabetic disorders that are appropriately applicable to human counterpart.^26,27^

In this study, we showed mild interstitial pneumonia and the degeneration of bronchiolar epithelium in diabetic lungs compared to the control rats. It seems that pathological changes in part, but not completely, may correlate with vascular inflammation. It is also suggested that continuously chronic glucose elevation in T2D altered normal function of type 2 pneumocytes and surfactant production, contributing to atelectasis and pulmonary tissue collapse which were not observed in our study.^3^

In this regard, both proteomic and genomic analyses showed the increase of TNF-α, IL-10 suppression, and up-regulation of ICAM-1 and VCAM-1. The induction of TNF-α *per se*disrupts blood-alveolar barrier integrity and promotes continuous immune cell extravasation.^3,28,29^ In a study, it was demonstrated that the ablation of TNF-α via genetic approaches prohibited the recruitment and infiltration of immune cells toward the lungs in the mice model of acute lung inflammation.^30^

Previous data showed that the accumulation of TNF-α increased the levels of endothelial adhesion molecules to promote immune cell recruitment in host tissues.^28,31^ TNF-α has the potential to weaken cell-to-cell connection while increase cell and plasma leakage to the extra-vascular microenvironment, leading to the formation of exudates in air sacs.^3,30^ In the support of this notion, the overproduction of NO in the diabetic pulmonary niche represented nitrosative stress and possible vasodilatation that accelerate continuous neutrophil recruitment from the blood system to the pulmonary microenvironment.^32^

Due to direct exposure of lungs and airway conduits to the environment, it seems that the progression of chronic metabolic diseases such as T2D abrogates the normal function of epithelium and innate defense system, leading to purulent and non-purulent neutrophilic leukocytosis.^33^

There are some limitations to this study. We did not perform specific pathological examinations such as periodic acid–Schiff stain to explore the content of mucoprotein and glycoproteins in the diabetic niche. Investigating spirometric analysis could help us forecast the physiological function of diabetic lungs compared to the control.

## Conclusion

In conclusion, the current study highlighted the importance of T2D in the induction of pathological changes and structural remodeling in the pulmonary niche like other tissues. The promotion of micro- and macro-angiopathies is touted as one of the critical factors involved in the diabetes-related pulmonary injury.

## Ethical Issues

Ethics permission for the study was received from the local ethical committee of Tabriz University of Medical Sciences (No: TBZMED. REC.1398.234).

## Conflict of Interest

Authors declared no conflict of interest.

## Acknowledgments

We gratefully thank the Vice-chancellor for research affairs of the Tabriz University of Medical Science for the financial support (No: TBZMED. REC.1398.234).

## Supplementary Materials


Supplementary file 1 contains Figure S1.
