# Supplementary figures and images for: Type 2 Diabetes Mellitus Provokes Rat Immune Cells Recruitment into the Pulmonary Niche by Up-regulation of Endothelial Adhesion Molecules
Source: Adv Pharm Bull. 2020 Oct 19;12(1):176–82. doi: 10.34172/apb.2022.019 (PMC9012922; doi:10.34172/apb.2022.019)

## Supplementary file 1

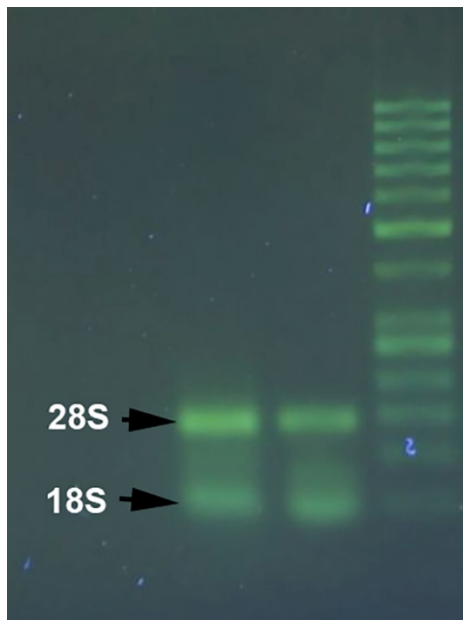

**Figure S1.** Evaluation of isolated RNA using 1% gel agarose electrophoresis

Supplement: Supplementary file 1 — contains Figure S1. [file apb-12-176-s001.pdf]
